# Comparison of the diagnostic capabilities of tNGS and mNGS for pathogens causing lower respiratory tract infections: a prospective observational study

**DOI:** 10.3389/fcimb.2025.1578939

**Published:** 2025-06-10

**Authors:** Yuchen Ding, Chuwei Jing, Jiachen Wei, Danni Wang, Wen Li, Mingyue Wang, Ji Zhou, Qian Qian, Wenkui Sun

**Affiliations:** ^1^ Department of Respiratory and Critical Care Medicine, The First Affiliated Hospital with Nanjing Medical University, Nanjing, Jiangsu, China; ^2^ Department of Clinical Medicine, Jiangsu Health Vocational College, Nanjing, Jiangsu, China

**Keywords:** lower respiratory tract infection, NGS - next generation sequencing, mNGS (metagenomic next-generation sequencing), targeted next generation sequencing (tNGS), diagnosis

## Abstract

**Aims:**

Pathogens in lower respiratory tract infections(LRTI) are complex. Conventional microbiological testings(CMTs) are time-consuming and inaccurate. mNGS is widely used to overcome these issues. tNGS, as an emerging NGS technology, has uncertain diagnostic efficacy.

**Materials and methods:**

136 suspected LRTI patients were included from January 2022 to February 2024 from the Department of Respiratory and Critical Care Medicine at Jiangsu Province People’s Hospital,China.We simultaneously submitted the bronchoalveolar lavage fluids (BALFs) for mNGS, tNGS and conventional microbial testing (CMTs) and compared the pathogen diagnostic efficacy of mNGS, and tNGS.

**Results:**

A total of 136 patients were included, and there was no statistically significant difference in the detection sensitivity(74.75% VS 78.64%, p>0,05) and specificity(81.82% vs 93,94%,p>0.05) between mNGS and tNGS. However, tNGS has a higher sensitivity(27.94% vs 17.65%,p=0.043)and specificity(88.78% vs 84.82%,p=0.048) for fungi. According to our diagnostic criteria, tNGS successfully identified 3 cases of Pneumocystis jirovecii(P. jirovecii) individually. In addition, both tNGS and mNGS detected chlamydia psittaci whereas CMTs were unable to detect it.

**Conclusions:**

tNGS demonstrates diagnostic efficacy for pathogens in lower respiratory tract infections that is comparable to mNGS. However, tNGS has specific advantages in the detection of fungi. Considering the cost-effectiveness of tNGS, it is recommended to implement tNGS clinically for patients with lower respiratory tract infections.

## 1 Introduction

Lower respiratory tract infections (LRTIs) continue to be one of leading causes of disability and death globally. The detection of pathogenic microorganisms causing infectious diseases has always been a clinical focus (Diseases and Injuries, 2020). Early and accurate identification of the etiology of LRTI is essential for effective pathogen targeted therapy (Xu et al., 2023). Traditional pathogen detection technologies face limitations, such as low culture positivity, long detection time and difficulty in detecting fastidious bacteria (Fu et al., 2022). In addition, the sensitivity of detection decreases for patients receiving antibiotic treatment.

Next-generation sequencing (NGS) allows for high-throughput, massively parallel sequencing of thousands to billions of DNA fragments independently and simultaneously (Mitchell and Simner, 2019). Currently, metagenomics NGS (mNGS) has been widely applied in clinic (van Dijk et al., 2014). It can detect all nucleic acid sequences in a sample through a single sequencing process, and identify the species of pathogenic microorganisms while detecting the pathogens. Theoretically, almost all potential pathogens, including viruses, bacteria, fungi and parasites, can be accurately identified in a single measurement (Ge et al., 2021). This solves the disadvantage of PCR and other technologies requiring pre-set pathogens. However, there are still challenges in the application of mNGS in lower respiratory tract infections. Such as complex manual operations, complex data analysis, and high costs (Diao et al., 2022). In addition, the interference of human source sequences cannot be ignored. About 90% reads sequenced by mNGS in bronchoalveolar lavage fluids (BALFs) are host-derived (Liang M. et al., 2022), which can lead to errors in the results. Moreover, mNGS cannot detect DNA and RNA simultaneously. For the detection of RNA viruses, additional RNA sequencing is required, which incurs higher costs. To address these issues, the introduction of targeted NGS (tNGS) has technologically improved some of the deficiencies of NGS in pathogen diagnostics.

tNGS is a molecular detection method based on target amplification and high-throughput sequencing technologies. Its detection sensitivity is unaffected by the human genome and background microbiota. It also has advantages such as low detection cost, low sample requirements, easy standardization of workflow, and simultaneous detection of DNA and RNA pathogens. In a recently published retrospective study, authors have reported that tNGS demonstrated comparable overall pathogen yield rate with mNGS, in patient with LRTI, and implying the preferential use of tNGS in clinical management and diagnosis of patients with LRTI. (Li et al., 2025). However, the detection efficacy for different types of pathogens was not emphasized. We conducted this prospective study to further understand the detection efficacy of tNGS for different pathogens. And, we also attempt to explore the efficacy of tNGS in detecting resistance genes.

## 2 Methods

### 2.1 Study design

The study prospectively included all suspected LRTI patients admitted to the Department of Respiratory Medicine, Jiangsu Provincial People’s Hospital from January 2022 to February 2024. This study was approved by the institutional review board. Suspected lower respiratory tract infection diagnostic criteria:(i).Lung imaging showing a new or progressive infiltrate, consolidation, or ground-glass opacity; (ii).Any of the following four criteria,①.Body temperature >37°C; ②. At least one respiratory symptom among cough, sputum production, dyspnea, chest pain, or altered breath sounds at auscultation; ③. Peripheral blood leukocyte count >10×10^9 or <4×10^9.An initial diagnosis of lower respiratory tract infection was made based on these criteria. The final diagnosis of lower respiratory tract infection is a composite standard. Based on the patient’s imaging characteristics, pathogen results (including NGS results), and treatment outcomes, the final diagnosis is made by the clinicians (Liang M. et al., 2022; Velly et al., 2023; Li et al., 2025).

### 2.2 Conventional microbiological tests

All enrolled patients underwent traditional laboratory tests, including BALF culture, 1,3-β-glucan synthase, galactomannan (GM), polymerase chain reaction(PCR), IgM(included *Chlamydia pneumoniae, M. pneumoniae, influenza virus, Epstein-Barr virus, Cytomegalovirus, influenza virus, epstein-barr virus, cytomegalovirus, adenovirus, parainfluenza virus, respiratory syncytial virus* Covid-19), and Tuberculosis-specific enzyme-linked immunospot assay(T-spot). The results of conventional microbiological tests(CMTs) were interpreted according to standard procedures (**Supplementary Table S1**). Some patients could not complete all tests due to reasons such as death or transfer to another facility.In our study, besides laboratory test positivity, the diagnosis of pneumonia and the responsible pathogens also requires two experienced clinicians(1.Wenkui Sun;2.Ji Zhou) independently to confirm the final diagnosis.

### 2.3 Data collection

For each enrolled patient, we documented demographic data, underlying conditions, laboratory test results(include serum and balf), tNGS and mNGS results, complications, and treatments during ICU hospitalization. Patient outcomes included ICU and in-hospital mortality rates, as well as ICU and hospital length of stay.

### 2.4 Sample collection

Erological tests were submitted within 24 hours of patient enrollment, including GM, tuberculosis PCR, influenza A/B PCR, respiratory virus IgM, and Epstein-Barr virus/cytomegalovirus PCR.All enrolled patients underwent bronchoscopy with bronchoalveolar lavage within 48 hours of admission.The bronchoalveolar lavage fluid was collected and simultaneously sent for tNGS and mNGS testing.Simultaneously, culture, G, GM, and T-spot were also tested.

### 2.5 Metagenomic next-generation sequencing

#### 2.5.1 Library construction and sequencing

DNA was extracted with a TIANamp Micro DNA Kit (TIANGEN BIOTECH, Art. No. DP316) following the manufacturer’s operational manual, and the total mass of extracted DNA, which must be more than 5 ng, was measured by Qubit dsDNA HS Assay Kits (Thermo Fisher Scientific, Art. No. Q32851). The DNA was digested into the appropriate length (200–300 bp) through a special fragment enzyme reaction, and the ends were filled with another enzyme. Meanwhile, phosphorylation was carried out at the 5′ end, and a Da tail was added at the 3′ end. Subsequently, the fragment DNA was connected with the adapter sequence under the action of DNA ligase, and purification beads were used to remove the splice dimers, redundant splices and residual reagents. The concentration of the constructed DNA library was determined by qPCR and should be more than 1 Nmol/L. Finally, all sample DNA libraries were mixed and sequenced with an Illumina NextSeq CN500 sequencer (SE strategy, read length=75). For each run, we used environment control sample to monitor microbial DNA signals arising from the background at the time of batch processing, and used different ID spike variants to monitor sample-to-sample contamination.

#### 2.5.2 Bioinformatics pipeline

Bcl2fastq software was used to split BCL sequence data into fastq-format files for each sample and the raw reads were quality filtered using fastp and kz software, including adapter contamination and low-quality and low-complexity reads. Next, after quality filtering, the clean reads were mapped to a curated human-related reference genome database containing hg38 and some non-human primer genome sequences using bowtie2. The remaining reads were aligned to the curated nonredundant bacterial, viral, fungal and parasite databases using SNAP. By annotating the mapped reads using an in-house program and counting the number of species-specific sequences, taxon profiles of the samples were generated.For the detected species, we further calculated the coverage of the species genome and the average sequencing depth of the coverage area using samtools and bedtools as additional indicators reflecting the reliability of the detected species. If the number of reads detected for a species was less than 3, we extracted the read sequences of the corresponding species that were aligned to the NT database for verification and kept the check-out results consistent with the verification. All species included in the curated pathogen reference databases were collected from books, such as the Manual of Clinical Microbiology, Diagnosis and Illustration of Clinical Microbiology, and NCBI RefSeq genome database. (12895 species of bacteria, 11120 species of viruses, 1582 species of fungi, 312 species of parasites, 177 species of mycobacteria, and 184 species of Chlamydiae/Rickettsiae).

#### 2.5.3 Interpretation of mNGS result

For all the bacteria originally detected, the obvious sequence alignment abnormalities (for the detected species, genome coverage <1% and depth >2) and background polluted bacteria (considered to be the exact background bacteria when the taxon-specific read number falls within the normal fluctuation range of historical statistical data compared with the negative controls) were first filtered out, and then pathogen data interpretation and pathogen positive determination were carried out as follows:(i)For Mycobacterium tuberculosis (M. tuberculosis), due to the difficulty of DNA extraction and low possibility for contamination, when at least 1 taxon-specific read was mapped to either the species or genus level, the result were considered as M. tuberculosis positive.(ii)For bacteria other than mycobacteria, viruses and parasites, the top 10 most abundant species within each category were selected, and the suspected pathogens were reported considering the clinical characteristics.

### 2.6 Targeted next-generation sequencing

#### 2.6.1 Library construction and sequencing

The library was prepared utilizing the Respiratory Pathogen Detection Kit, and a no template control was included to oversee the library construction and sequencing procedures. This process involved two rounds of PCR amplification. The sample nucleic acid and cDNA served as templates, and a panel of 153 microorganism-specific primers was chosen for ultra-multiplex PCR amplification to enrich the target pathogen sequences, encompassing bacteria, viruses, fungi, mycoplasma, and chlamydia. Following amplification, PCR products were purified using beads, then subjected to amplification with primers containing sequencing adapters and unique barcodes. The quality and quantity of the constructed library were assessed using the Qsep100 Bio-Fragment Analyzer (Bioptic, Taiwan, China) and Qubit 4.0 fluorometer (Thermo Scientific, Massachusetts, United States), respectively. Typically, the library fragments displayed sizes ranging from approximately 250 to 350 bp, with the library concentration being kept at a minimum of 0.5 ng/μl. The concentration of the pooled library was re-evaluated and then diluted to a final concentration of 1 nmol/L. Subsequently, 5 μl of the pooled library was combined with 5 μl of freshly prepared NaOH (0.1 mol/L). After a brief vortexing and centrifugation step, the library was incubated at room temperature for 5 min. The diluted and denatured library was subsequently sequenced on an Illumina MiniSeq platform utilizing a universal sequencing reagent kit (KS107-CXR, KingCreate, Guangzhou, China). On average, each library yielded approximately 0.1 million reads, with a sequencing read length of single-end 100 bp.

#### 2.6.2 Bioinformatics pipeline

Sequencing data were analyzed using the data management and analysis system (v3.7.2, KingCreate). The raw data underwent initial identification via the adapter. Reads with single-end lengths exceeding 50bp were retained, followed by low-quality filtering to retain reads with Q30>75%, ensuring high-quality data. The single-ended aligned reads were then compared using the Self-Building clinical pathogen database to determine the read count of specific amplification targets in each sample. The reference sequences used for read mapping was a database curated from different sources, including Genbank database, Refseq database, and Nucleotide database from NCBI.

#### 2.6.3 Interpretation of tNGS result

In line with the experimental principle of targeted amplification of microbial sequences using specific primers, the amplicon coverage and normalized read count of detected microorganisms within the sample constituted the primary interpretation indicators. To categorize a microorganism as a potential pathogen, the following criteria were established: (i) bacteria (excluding M. tuberculosis complex), fungi and atypical pathogen: amplicon coverage ≥50% and normalized read count ≥10; (ii) viruses: amplicon coverage ≥50% and normalized read count ≥3, or normalized read count ≥10; (iii) M. tuberculosis complex: normalized read count ≥1.

### 2.7 The interpretational approaches of mNGS and tNGS

Optimal thresholds were set up as listed here in order to identify true pathogens:

- Laboratory testing, which provides reported results after data quality control.
- *Burkholderia spp.*, *Ralstonia spp*., and *Delftia spp*. were considered as positive when relative abundance in genus level ≥ 80%, since they were regarded as the most common contamination genera in the lab, and had rarely been cultured or validated by specific polymerase chain reaction (PCR) as pathogens in a microbiology lab;
- For oral and respiratory tract colonizing microorganisms such as Streptococcus spp.(excluding Streptococcus pneumoniae), Haemophilus spp.(excluding Haemophilus influenzae). These pathogens need to meet the following criteria to be considered meaningful: i)excluding other pathogenic microorganisms. ii)relative abundance > 80% or sequence coverage 10 times higher than any other microorganism.
- Determine the optimal diagnostic cut-off point based on the ROC curve results:Bacteria:the relative abundance of mNGS ≥ 6.549%;the normalized read count of tNGS ≥ 3455;Fungi:the normalized read count of mNGS ≥ 470;the normalized read count of tNGS ≥2216;*M.tuberculosis* and *P. jirovecii*:normalized read count ≥1. Virus:included cases of COVID-19, influenza virus A, and influenza virus B.

Subsequently, two experienced independently conducted a comprehensive assessment of the patient’s clinical data to determine the presence of LRTI and the clinical relevance of potential pathogens in both mNGS and tNGS.

### 2.8 Statistical analysis

All clinical and laboratory data were analyzed using the non-parametric Mann-Whitney U test, the chi-squared test,and the independent-samples t-test. The sensitivities and specificities of microbiological tests were compared using the McNemar’s test for related proportions. A p-value < 0.05 was considered significant. All analyses were performed using the SPSS v26.0 software (IBM, Armonk, New York, USA).

## 3 Results

### 3.1 Samples

A total of 136 suspected LRTI patients were enrolled, including 97 males. The average age of the patients is 68 years, and the median APACHE II score is 19.97. Sixty-six patients are considered immunocompromised. The clinical and laboratory data for all cases are shown in **Table 1**.

**Table 1:** Characteristics of 136 patients with suspected LRTIs.

Characteristic	Value
demographic information	
Age (year), median (range)	68 (27-94)
Male, n (%)	97 (71.32%)
Immunocompromised population,n (%)	42 (30.88)
Open airway,n (%)	78 (57.35)
Serological data	
White blood cell count (10 9 /L), median (range)	10.81 (2.46-38.75)
Lymphocyte count (10 9 /L), median (range)	0.96 (0.11-5.63)
CRP (mg/L),range	103.85 (1.02-460)
PCT (ng/ml),range	2.52 (0.043-68.11)
Comorbidities	
hypertension,n (%)	51 (37.68)
diabetes,n (%)	31 (22.79)
tumours,n (%)	35 (25.74)
hepatic insufficiency,n (%)	58 (42.86)
renal insufficiency,n (%)	70 (44.12)
Others	
APACHE II score, median (range)	19.97 (7-36)
ICU LOS (days), median (range)	10.33 (5-59)

### 3.2 Pathogens

We diagnosed a total of 103/136 LRTI patients.Of the 103 patients, 66 patients had monomicrobial infections, while 37 patients had polymicrobial infections (including 31 patients with 2 pathogens and 6 patients with 3 pathogens). Sixty patients had bacterial infections (*Acinetobacter baumannii* in 23 cases, *Pseudomonas aeruginosa* in 16 cases, *klebsiella pneumoniae* in 12 cases), 36 patients had fungal infections (*Aspergillus* spp. In 17 cases, *P. jirovecii* in 5 cases), and 22 patients had viral infections (*influenza virus in 13 cases and Covid-19* in 4 cases). We also detected 5 cases of *M.tuberculosis* infection and 5 cases of *Chlamydia psittaci* infection. **Table 2**.

**Table 2:** The composition of pathogens.

Pathogen identification	Value^a^
Bacteria	
Acinetobacter baumannii	23
Pseudomonas aeruginosa	16
klebsiella pneumoniae	12
staphylococcus aureus	11
streptococcus pneumoniae	4
haemophilus influenzae	4
stenotrophomonas maltophilia strain	2
burkholderia cenocepacia	2
s.marcescens	2
nocardia	1
moraxella catarrhalis	1
Fungi	
Aspergillus spp	17
candida	15
Pneumocystis jirovecii	5
Virus	
Influenza A	12
parainfluenza virus	5
Covid-19	4
HSV1	2
βcoronavirus	1
Influenza B	1
Atypical pathogens	
M.tuberculosis	5
Chlamydia psittaci	5

a :The number of diagnosed pathogens.

### 3.3 Comparison of diagnostic efficacy between mNGS and tNGS

The overall diagnostic positivity for mNGS and tNGS were both 61.09%, exceeding the results of CMTs(50.74%). tNGS compared to mNGS exhibits higher sensitivity(78.64% vs 74.75%), specificity (93.94% vs 81.82%), positive predictive value(PPV, 97.59% vs 92.77%) and negative predictive value (NPV, 58.49% vs 50.94%), but not significantly (**Table 3**). Both methods demonstrated superior positivity, sensitivity, specificity, PPV and NPV. Furthermore, for the 5 patients definitively diagnosed with Chlamydia psittaci,both mNGS and tNGS detected it.

**Table 3:** Diagnostic performance of mNGS,tNGS and CMTs.

	Positivity%^a^	Sensitivity%	Specificity%	PPV%	NPV%
mNGS	61.09%	74.75%	81.82%	92.77%	50.94%
tNGS	61.09%	78.64%	93.94%	97.59%	58.49%
CMTs^b^	50.74%	57.28%	69.7%	85.51%	34.33%

a Positivity: the proportion of patients with positive results to the total number of patients.

b CMTs: conventional microbiological tests.

NPV: negative prediction value; PPV: positive prediction value.

tNGS and mNGS both diagnosed 5 cases of *M. tuberculosis*, which is consistent with our diagnostic results. Additionally, tNGS reported one false positive case. This patient had a previous diagnosis of *M. tuberculosis*, but had already recovered after anti-tuberculosis treatment.

### 3.4 Comparison of pathogens detected by mNGS and tNGS

**Figure 1:**
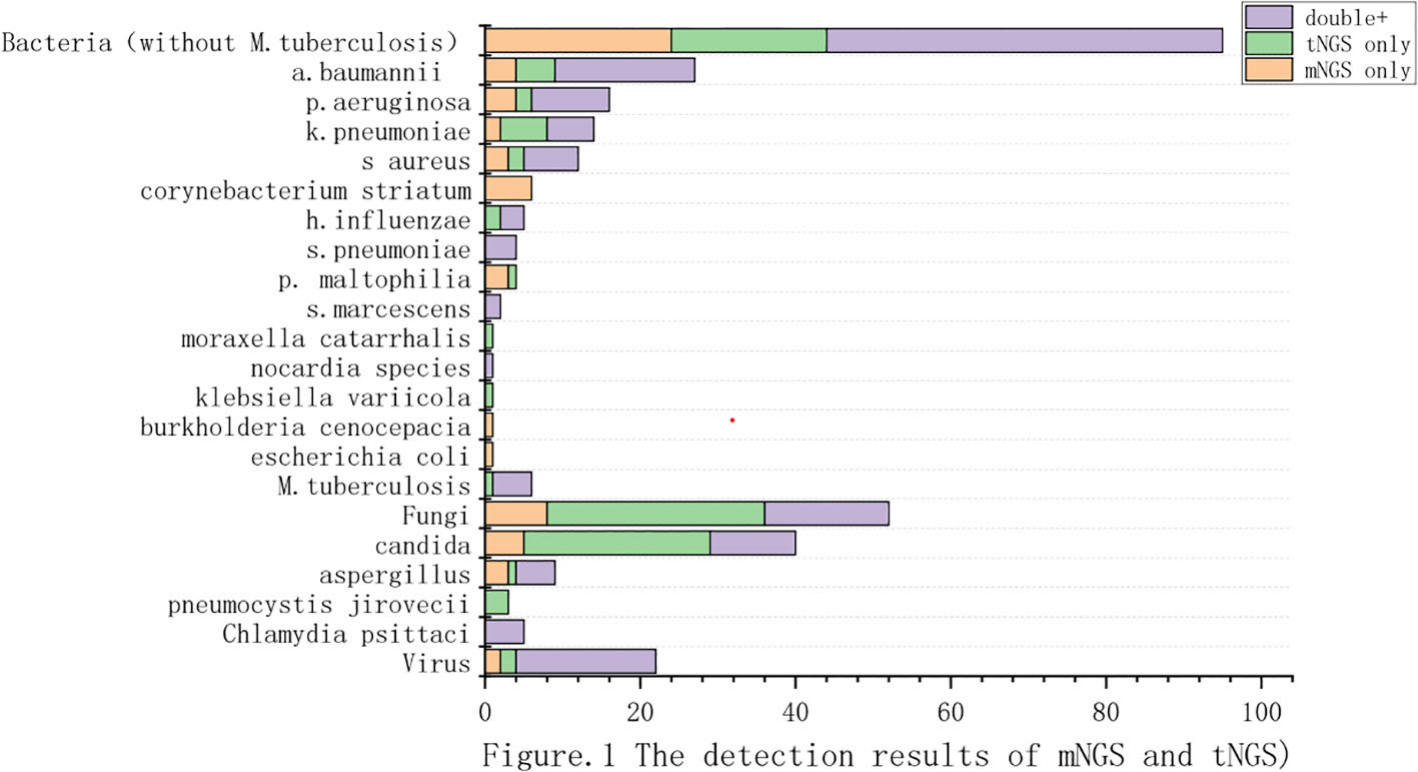
The detection results of mNGS and tNGS. (We compared the results of pathogen detection between tNGS and mNGS. The purple section indicates the pathogens detected by both methods. The green section represents the pathogens detected by tNGS but not by mNGS. The yellow section represents the pathogens detected exclusively by mNGS.).

In mNGS, the positivity of pathogens detection reached 61.09%(83/136). We detected 75 strains of bacteria in 12 species, 24 strains of fungi in 5 species, 5 cases of *M.tuberculosis*, and 5 cases of atypical pathogens in total. Among the detected bacteria, the most commonly detected were *Acinetobacter baumannii*(n=22), followed by *Pseudomonas aeruginosa*(n=14), *Staphylococcus aureus*(n=10), *Klebsiella pneumoniae*(n=8) and *Corynebacterium striatum*(n=6). The 5 fungi detected were *candida albicans*(n=12), *aspergillus fumigatus*(n=7), *candida tropicalis*(n=3), *aspergillus flavus*(n=1) and *candida parapsilosis*(n=1). In addition, 5 cases of *M.tuberculosis* and 5 cases of *Chlamydia psittaci* were detected. **Figure 1**.

In tNGS, the positivity of pathogens detection also reached 61.09%(83/136).A total of 71 bacteria in 11 species were detected,including *Acinetobacter baumannii*(23/136), *Klebsiella pneumoniae*(12/136), *Pseudomonas aeruginosa*(12/136), *Staphylococcus aureus*(9/136), *Haemophilus influenzae*(5/136). In terms of fungal identification, we detected Candida spp. (35/136) and *Aspergillus spp*.(6/136). We independently identified *P. jirovecii* (n=3). In addition, 6 cases of *M.tuberculosis* and 5 cases of *Chlamydia psittaci* were detected.

### 3.5 Comparison of the diagnostic efficacy of mNGS and tNGS for bacteria and fungi

A total of 60 bacterial infections (excluding *M.tuberculosis*) were identified. The Specificity for mNGS was 89.47%, while for tNGS it was 97.37%(p=0.05). Depite of this,there were no statistically significant differences between mNGS and tNGS in detecting bacteria.

Analysis of 36 fungal infection cases revealed that the detection of mNGS was significantly lower than that of tNGS(17.65% vs 27.94%,p=0.043). The specificity of tNGS was also significantly higher than that of the mNGS (88.78% vs 84.82%, p< 0.05). These results were displayed in **Table 4**.

**Table 4:** Comparison of the diagnostic efficacy of mNGS and tNGS for bacteria and fungi.

	Positivity%^a^		Sensitivity%		Specificity%		PPV%		NPV%	
	Bacteria	Fungi	Bacteria	Fungi	Bacteria	Fungi	Bacteria	Fungi	Bacteria	Fungi
mNGS	37.5%	17.65%	85%	52.78%	89.47%	84.82%	86.44%	79.17%	88.31%	84.82%
tNGS	36.76%	27.94%	83.33%	69.44%	97.37%	88.78%	96.15%	65.79%	88.10%	88.78%
P	0.388	0.043	0.803	0.147	0.05	0.048	0.1	0.258	0.966	0.4

a Positivity: the proportion of patients with positive results to the total number of patients.

NPV: negative prediction value; PPV: positive prediction value.

### 3.6 Concordance between mNGS and tNGS for pathogen detection

**Figure 2:**
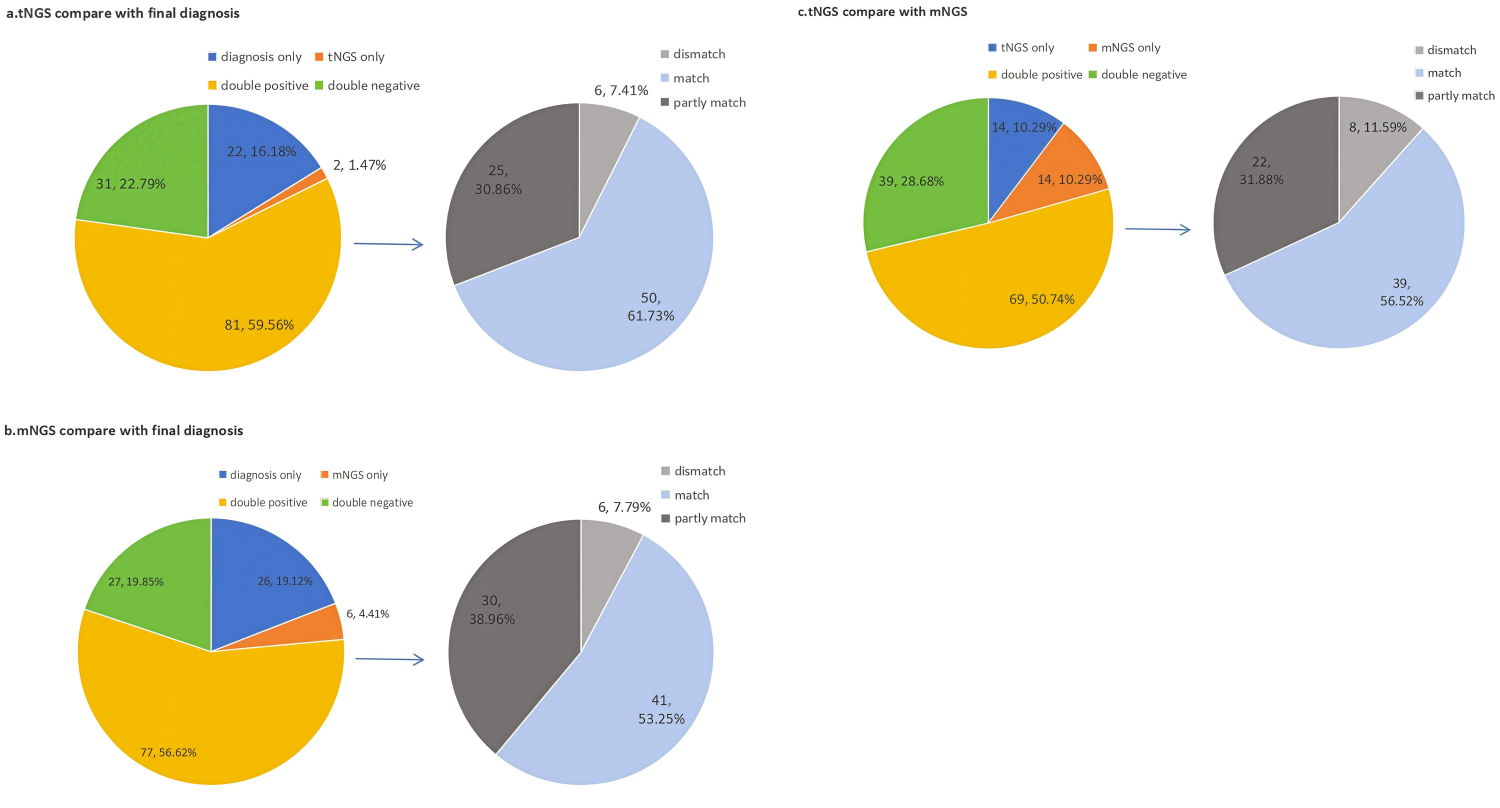
Concordance between final diagnosis. mNGS and tNGS for pathogen detection. (We compared the consistency between tNGS, mNGS, and final diagnosis. and further analyzed the subset of double positive results. (a) Comparison of consistency between tNGS and final diagnosis. (b)Comparison of consistency between mNGS andfinal diagnosis.; (c) Comparison of consistency between mNGS and tNGS.).

In the 136 samples of BALFs, the tNGS and final diagnosis identified 81 cases (59.56%) as positive. Of them, 50 cases (61.73%) were in perfect agreement, 25 cases(30.86%) had some degree of concordance (one or more detected pathogens were similar) and 6 cases (7.41%) were completely different.(**Figure 2a**).

In addition,77 cases(56.62%) cases were identified positive by both mNGS and final diagnosis. Of them, 41 cases(53.25%) were in perfect agreement, 30 cases(38.96%) had some degree of concordance (one or more detected pathogens were similar) and 6 cases (7.79%) were completely different. (**Figure 2b**).

Finally,we compared the detection results between tNGS and mNGS. Out of 136 samples, 69 samples(50.74%) showed double positive results, and 39 samples(28.68%) showed double negative results.Beside of this,tNGS and mNGS each individually detected 13 cases. The concordance rate between tNGS and final diagnosis was higher (χ² =16786, P < 0.05), which was more in line with clinical practice. (**Figure 2c**).

### 3.7 Detection of viruses by tNGS and mNGS

We conducted Covid-19 PCR testing on all patients. 4 cases of Covid-19 patients were confirmed. Both mNGS and tNGS diagnosed 3 cases. We also conducted viral IgM testing on 112 patients, including *adenovirus,influenza A virus, influenza B virus, parainfluenza virus and respiratory syncytial virus*. The detection of influenza viruses was relatively high, with 21 cases of influenza A virus and 24 cases of influenza B virus detected. Among them, 22 patients were highly suspected of influenza virus infection, so we performed influenza virus PCR testing. The results of the influenza virus PCR showed 2 positive cases of influenza A virus and 1 positive case of influenza B virus. Both mNGS and tNGS were performed on all patients, detecting 19 cases and 18 cases of influenza virus, respectively. In addition, we conducted EBV IgM testing on 34 patients and CMV IgM testing on 61 patients. 15 patients were considered to have EBV infection, while 6 patients were considered to have CMV infection.**Table 5**.

**Table 5:** The methods and results of the virus detection.

	Method	Specimen	Samples	Positive results^a^	tNGS^b^	mNGS^b^
COVID-19	PCR	Throat swab	136	4	3	3
EBV	PCR	serum	34	15	32	36
CMV	PCR	serum	61	6	30	41
Adenovirus	PCR	serum	26	0	2	2
	IgM	serum	112	3		
Influenza A	PCR	Throat swab	22	2	17	17
	IgM	serum	112	21		
InfluenzaB	PCR	Throat swab	22	1	2	1
	IgM	serum	112	24		
Parainfluenza virus	IgM	serum	112	3	5	5
RSV	IgM	serum	112	2	5	3

a : The positive results of the virus detection.

b : Both tNGS and mNGS were performed on all patients.

### 3.8 Concordance between drug resistance genes and phenotypic resistance

**Figure 3:**
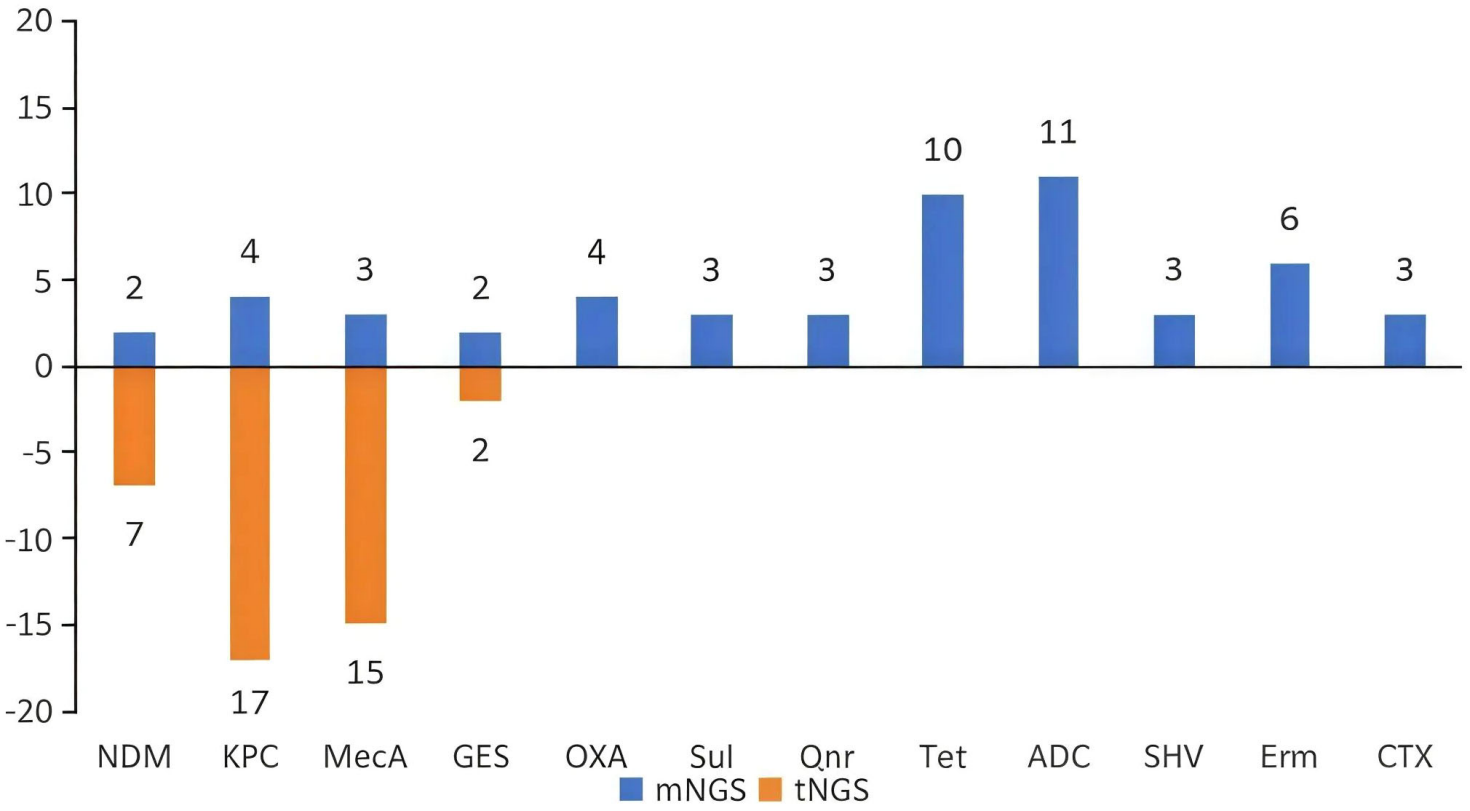
Results of drug-resistant gene detection using tNGS and mNGS. (The resistance gene profile of tNGS included four types of resistance genes. mNGS detected more resistance genetypes).

tNGS identified 4 types of drug-resistant genes: 17 cases of KPC, 15 cases of MecA, 7 cases of ND, and 2 cases of GES. In addition, mNGS identified other drug-resistant gene types, including OXA, Van, Tet, and Erm. **Figure 3**.

mNGS detected drug-resistant genes in 33 samples, while tNGS detected them in 57 samples.The detection of drug-resistant genes by mNGS(42.64%) is significantly higher than that of tNGS(24.26%). In cases with limited drug sensitivity test results, tNGS predicted drug-resistant genes in 9/16 samples(56.25%), which is consistent with the phenotypic susceptibility results. And mNGS exhibited a concordance rate of 83.87% in 31 samples(26/31). **Table 6**.

**Table 6:** Detection of resistance genes in tNGS/Mngs.

	tNGS	mNGS
Samples	33	57
positivity^a^	24.26%(33/136)	42.64%(57/136)
AST^b^	16	31
Agreements	56.25%(9/16)	83.87%(26/31)

a : tNGS detected 33 cases of antibiotic resistance genes, while mNGS detected 57 cases.

b : Among the samples of resistance genes detected by tNGS, 9 cases had drug sensitivity results, while 26 cases were detected by mNGS.

### 3.9 Application of NGS in clinical practice

To illustrate the clinical performance of tNGS and mNGS, we have presented two case examples. Both cases had no pathogens detected by CMTs. In Case A, both tNGS and mNGS detected *Chlamydia psittaci*. After adding omadacycline, the patient’s symptoms did not show significant improvement. However, tNGS additionally detected *Aspergillus*. A bronchoscopy biopsy was performed again on Day 6, revealing active bleeding in the left lung. Invasive pulmonary aspergillosis was considered. Therefore, we initiated antifungal treatment, and the patient improved.

**Figure 4:**
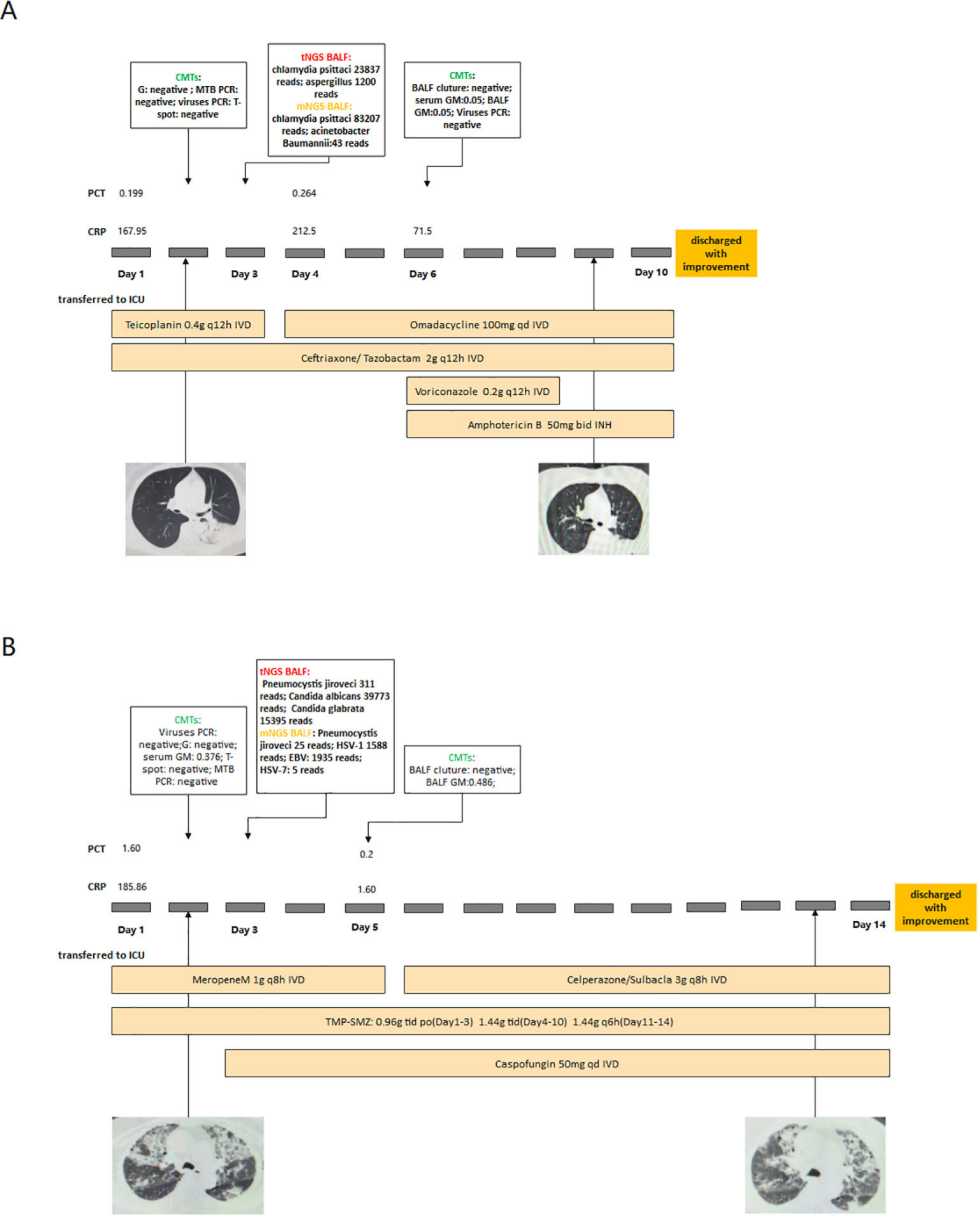
Two cases of NGS guided clinical treatment (Case **(A)** Chlamydia psittaci infection combined with Aspergillus infection. Case **(B)** PJP combined with Candida infection).

In Case B, both tNGS and mNGS detected the pathogen of *P. jirovecii*. The patient had interstitial lung disease and was concurrently infected with *Candida albicans*. tNGS detected *Candida* exclusively, while mNGS did not identify it (**Figure 4**).

## 4 Discussion

Research shows that the detection capabilities of tNGS and mNGS in BALFs are comparable (Li et al., 2022), but no further analysis was conducted. Therefore, we attempted to further discuss the diagnostic capabilities of mNGS and tNGS based on clinical results. Under inspiration by other studies (Huang et al., 2020; Liang et al., 2024), an objective interpretation criterion based on reads and relative abundance of NGS results was established. The cutoff for detecting bacteria is relative abundance in mNGS, and is reads in tNGS. The cutoff of fungi can be detected by reads. Through this method, we have minimized the influence of subjective factors and effectively distinguished between colonizing organisms and pathogenic bacteria.

Obviously, both mNGS and tNGS showed superior detection performance than CMTs. Nevertheless, the tNGS results matched the CMTs at the species level better than the mNGS results (61.73% vs. 53.25%), which is mainly attributed to technological differences. tNGS can disregard the influence of human host sequences, which reduces the work amount required for data processing (Huang et al., 2023). tNGS can amplify near-complete genomic sequences directly from low nucleic acid clinical samples (Wylie et al., 2015; Paskey et al., 2024), which increases its sensitivity. The results of mNGS are complex (Gu et al., 2019), as only a small proportion (typically <1%) of reads in mNGS are non-human, of which only a subset may correspond to potential pathogens (Chiu and Miller, 2019). In comparison, tNGS can detect some pathogens that were disregarded by mNGS. In addition, mNGS detects a more diverse spectrum of pathogens, while tNGS is limited by the pathgen panel (Chiu and Miller, 2019). Most studies using tNGS panels involve hundreds to thousands of clinically important or relevant pathogens (Sun et al., 2024; Wei et al., 2024). Though mNGS theoretically can identify all pathogenic microorganisms, it is limited by the content of the library (Naccache et al., 2014).

tNGS and mNGS perform differently in detecting various pathogens. Overall, mNGS is more likely to detect contaminating bacteria, such as oral flora. tNGS can improve enrichment efficiency, reduce contamination, and is expected to address this issue in the future (Yin et al., 2024). tNGS shows higher detection and sensitivity for fungi, which have not been confirmed in, probably due to incomplete destruction of cell wall (Babady et al., 2024; Huang et al., 2024; Wei et al., 2024). Fungi have thick cell walls and require cell wall disruption for DNA extraction (Naccache et al., 2014; Li et al., 2021). The fragmented nucleic acids as-extracted may be below the detection threshold of mNGS, but can be sequenced by tNGS through amplification. tNGS has greater potential for diagnosing fungi by improving technological capabilities. A common trait among viruses, especially RNA viruses, is their elevated ability to generate genetic variability (Quer et al., 2022). mNGS theoretically can identify all mutations, including unknown viruses, but this ability shall be confirmed by further research (Hodinka, 2016; Gauthier et al., 2023). Unlike PCR methods that only detect a limited number of predefined targets. Targeted enrichment-based NGS technology is effective in detecting clinical samples with relatively low viral loads (Lee et al., 2022). Our research shows that mNGS detected more viruses than tNGS. But, further research is needed to determine whether the detected virus is pathogenic.

The diagnosis of fastidious pathgens can be very challenging, as fastidious bacteria are difficult to culture, have long incubation time, and low positivity (Lagier et al., 2015). Clinically, these challenges can lead to antibiotic misuse and delayed treatment.The detection of *M.tuberculosis* mainly relies on Xpert (Steingart et al., 2014), and rapid diagnosis of *Chlamydia psittaci* currently depends on PCR (Vande Weygaerde et al., 2018), which yet cannot guarantee detection efficiency. The detection efficacy of mNGS for *mycobacterium tuberculosis* and *Chlamydia psittaci* has been confirmed (Vande Weygaerde et al., 2018; Zhou et al., 2019). Both of these detection methods require a prior suspicion of the pathogens and cannot be used routinely as clinical diagnostic methods. NGS does not require a preconceived notion of the pathogen and can partially address the delayed diagnosis.In our study, both tNGS and mNGS detected two pathogens with 100% accuracy. These achievements offer a major advantage in clinical diagnosis and prognosis of fastidious pathgeons.

Attention shall also be paid to the interpretation of results. Among the fungi identified as negative, mNGS detected *P. jirovecii* most frequently, while tNGS ranked it second, following *Candida albicans*. There is no universally accepted diagnostic method for *P. jirovecii*, and the existing methods have difficulty distinguishing between colonized and infected patients (Kolbrink et al., 2022). In our study, 5 cases of *P. jirovecii* were diagnosed. tNGS detected 3 cases as positive (3/15), which all reports high reads per kilobase per million mapped reads (RPKM), but the mNGS results were all negative (0/13). tNGS enriches sequences of *P. jirovecii*, providing a potential means for distinguishing the pathogenicity of *P. jirovecii* through differential RPKMs. Nevertheless, there is no unified standard for interpreting NGS results (Simner et al., 2018). Previous studies have interpreted results based on laboratory results, relative abundance, SDSMRN (number of unique reads of standardized species), culture results, and coverage (Miao et al., 2018; Li et al., 2020; Yang et al., 2022). These methods are influenced by various factors in clinical practice,such as lung microbiota, potential synergistic interactions between pathogens, and the release of DNA from dead pathogens (Semenec et al., 2023). We referred to previous studies to distinguish between opportunistic pathogens and colonizing organisms, and thought all infecting pathogens should have database reports (Flurin et al., 2021; Liang et al., 2024). The optimal diagnostic cutoff value was determined using parameters from the NGS report (readst, relative abundance, coverage). For common contaminants such as *Burkholderia spp., Ralstonia spp., and Delftia spp.*, the relative abundance should be larger than 80%. Based on these criteria, we established standards for clinical interpretation.

We also compared the detection capabilities between tNGS and mNGS in detecting drug-resistant genes. tNGS requires a pre-set panel of drug-resistant genes, and our panel includes 15 genotypes. In comparison, mNGS is an unbiased detection method that can identify all potential drug-resistant genes. Our results show that mNGS is over tNGS in detecting resistance genes and achieves consistency with drug susceptibility results. This limitation restricts the application of tNGS, but may be improved by including more drug-resistant gene profiles.

Currently, the use of the NGS technology in drug resistance detection is challenging. At the technical level, mNGS is limited by the relatively short nucleic acid read sequences (less than 300 bp), making it difficult to obtain complete sequences of drug-resistant genes (Liang WY. et al., 2022). Targeted or enrichment-based NGS methods may be a potential solution, but lack relevant research in this area. Furthermore, research has found poor consistency between antimicrobial resistance detection results and phenotypic AST (Gaston et al., 2022). In our study, tNGS and mNGS detected 7 and 5 cases, respectively, where the genotype was inconsistent with the phenotypic resistance profile. The reason for this inconsistency may be that gene transfer between bacterial species and the coordinated expression of multiple genes contribute to a specific resistance phenotype (Su et al., 2019). Therefore, in samples with high-abundance single microorganism pathogens, mNGS can more accurately detect antimicrobial resistance (AMR) genes (Rodino and Simner, 2024). In complex environments, mNGS detects multiple AMR genes, which mostly originate from non-pathogenic microorganisms, thus posing challenges for clinical interpretation. Targeted capture or enrichment strategies can improve detection sensitivity, but most of the AMR genes detected in targeted workflows are unrelated to the detected pathogens, because of the misalignment of resistance genes with pathogens (Gaston et al., 2022). Overall, in LRTIs, neither mNGS nor tNGS can completely replace traditional antimicrobial susceptibility testing.

## 5 Limitation

This study also has certain limitations. Firstly, our study samples were included from a single institution, and the testing results may be influenced by environmental and laboratory conditions. Therefore, our conclusions may not be applicable to other regions. Additionally, our tNGS pathogen profile includes only 153 pathogens associated with LRTI, and thus we cannot predict the performance of tNGS in detecting certain rare pathogens. Moreover, the results of both mNGS and tNGS are complex. Although we have established a relatively objective interpretation method, it may not be applicable to other NGS platforms. Finally, our tNGS panel just included 15 genotypes, which cannot meet the practical needs of clinical settings. Therefore, it is necessary to expand the antibiotic resistance gene profile and conduct further research.

## 6 Conclusions

tNGS shows capabilities that are not inferior to mNGS in detecting LRTI, and tNGS has greater potential for detecting fungi. Considering the cost advantages of tNGS, we recommend prioritizing its use for LRTI. Moreover, the limited antibiotic resistance gene profile of tNGS restricts its clinical application compared to mNGS. In the future, we need more clinical studies to explore the application of tNGS in different pathogens and types of infections. Additionally, expanding the antibiotic resistance gene profile of tNGS will contribute to its clinical application.

## Data Availability

The raw data supporting the conclusions of this article will be made available by the authors, without undue reservation.
