# Supplementary material for: Comparison of the diagnostic capabilities of tNGS and mNGS for pathogens causing lower respiratory tract infections: a prospective observational study
Source: Front Cell Infect Microbiol. 2025 Jun 10;15:1578939. doi: 10.3389/fcimb.2025.1578939 (PMC12185458; doi:10.3389/fcimb.2025.1578939)
Supplement: Supplementary file 1 [file Table1.docx]

**Supplementary Content**

**Figure S1.**

The workflow of the research

**Table S1**

Interpretation criteria for conventional microbiological tests(CMTs)

**Data 1.**

Relevant data of the diagnostic model.

**Figure S1.The workflow of the research**

**
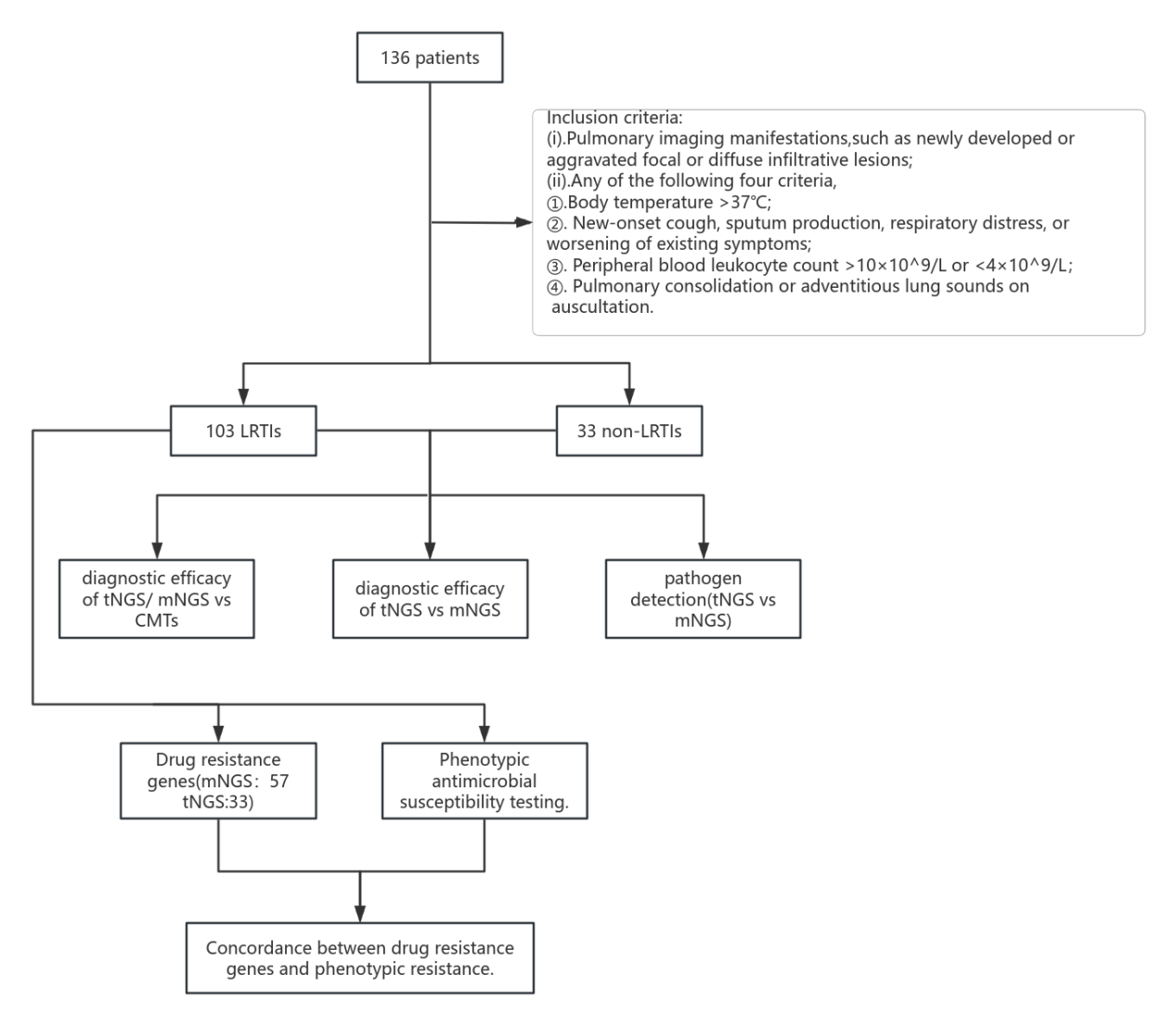
**

**Table S1** Interpretation criteria for conventional microbiological tests(CMTs)

| Pathogen | Definition of Clinically Significant Microbes |
| --- | --- |
| Bacteria | Moderate to heavy (++——+++)growths of bacteria with few epithelial cells seen on Gram stain examination (< 10 per high high-power field) from BALF; oral commensal organisms are considered as contaminants unless they are deemed significant by the managing physiciancian |
| *Legionella*, *Mycoplasma*, *Chlamydia*  *streptococcus pneumoniae* | Positive IgM  deemed significant by the managing physician  Positive *streptococcus pneumoniae*antigen test in urine specimen, or culture from BALF |
| *Mycobacterium tuberculosis*or NTM | Positive culture, or PCR test for *Mycobacterium tuberculosis*or  from Blood  T-spot positive deemed significant by the managing physician |
| Fungi |  |
| *Aspergillus*spp. | Positive culture from BALF, or BALF/serum GM > 1.0, or serum GM > 0.7 and BALF GM>0.8 |
| Fungi other than *Aspergillus*spp. | Positive culture for fungi other than *Aspergillus*spp. from BALF; *Candida*spp. are disregarded unless they are deemed significant by the managing physician |
| *Pneumocystis jirovecii* | Detection of cyst of *Pneumocyst*is by Gomori methenamine stain, or positive culture from BALF |
| *Cryptococcus* | Positive culture or positive antigen test for *Cryptococcus*from BALF; visualization of capsule by India ink stain in blood |
| Viruses |  |
| EB，CMV | Positive PCR from blood |
| Viruse other than EB and CMV | positive IgM  deemed significant by the managing physician |

**Data 1.Relevant data of the diagnostic model.**

A.bacterial detection in mNGS(the relative abundance)

| AUC | 95%cl | cutoff | Youden's index |
| --- | --- | --- | --- |
| 0.906 | 0.862-0.951 | 6.549 | 0.701 |

B.fungi detection in mNGS(the normalized read count))

| AUC | 95%cl | cutoff | Youden's index |
| --- | --- | --- | --- |
| 0.670 | 0.535-0.805 | 470 | 0.361 |

C.bacterial detection in tNGS(the normalized read count)

| AUC | 95%cl | cutoff | Youden's index |
| --- | --- | --- | --- |
| 0.946 | 0.915-0.978 | 3455 | 0.777 |

D.fungi detection in tNGS(the normalized read count))

| AUC | 95%cl | cutoff | Youden's index |
| --- | --- | --- | --- |
| 0.766 | 0.671-0.861 | 2216 | 0.455 |

We make positive determinations based on the relative abundance, normalized sequence counts, and coverage of NGS data.And we plotted the ROC curve.The final results show that the diagnosis of fungi through normalized sequence counts in both mNGS and tNGS is the most optimal (mNGS: AUC=0.670 , tNGS：AUC=0.766).Bacterial detection by mNGS is classified based on relative abundance（AUC=0.906）, while tNGS based on normalized sequence counts（AUC=0.946）.
